# Economic Losses from Premature Under-Five Mortality due to Diarrhoeal Diseases in South Asia: Trends, Aetiologies, and Key Risk Factors

**DOI:** 10.1007/s44197-026-00598-9

**Published:** 2026-07-03

**Authors:** Bijaya Kumar Padhi, Manya Soni, Vijay Kumar, Amogh Verma, Prajnasini Satapathy, Sorabh Lakhanpal, Rekha Arcot, Do-Youn Lee, Prakasini Satapathy

**Affiliations:** 1 Department of Community Medicine, School of Public Health, Postgraduate Institute of Medical Education and Research, Chandigarh, 160012 India; 2 Noida Institute of Engineering & Technology (Pharmacy Institute, Plot No.19, Knowledge Park-II, Greater Noida, India; 3 Centre for Promotion of Research, Graphic Era Deemed University, Dehradun, India; 4 Graphic Era Hill University, Dehradun, India; 5 Centre for Research Impact & Outcome, Chitkara College of Pharmacy, Chitkara University, Rajpura, 140401 Punjab India; 6 Malla Reddy Instiute of Medical Sciences, Malla Reddy Vishwavidyapeeth, Suraram, Hyderabad, 500055 Telangana India; 7 School of Pharmaceutical Sciences, Lovely Professional University, Phagwara, India; 8 Department of Surgery, Hospital and Research Centre, Dr. D. Y. Patil Medical College, Dr. D. Y. Patil Vidyapeeth (Deemed-to-be-University), Pimpri, Pune, 411018 Maharashtra India; 9 Kookmin University, Seoul, 02707 South Korea; 10 School of Medical Sciences and Research, Sharda University, Greater Noida, India

**Keywords:** Under-five mortality, Diarrhoeal diseases, Human capital approach, Economic burden, South Asia

## Abstract

**Background:**

Diarrhoeal diseases remain a major cause of under-five mortality in South Asia despite global health progress. This study quantifies the economic losses due to premature under-five deaths from diarrhoeal diseases, analyzing trends, aetiologies, and risk factors across five South Asian countries between 2010 and 2023.

**Methods:**

Using data from the datasets published by the Institute for Health Metrics and Evaluation (IHME) and World Bank indicators, the study applied the human capital approach to estimate the present value of lost future labour income associated with diarrhoeal deaths among children under five. Losses were estimated in both purchasing power parity (PPP)-adjusted and nominal U.S. dollars to reflect real productivity losses and fiscal implications, respectively.

**Results:**

While age-standardized mortality and DALY rates declined significantly across South Asia (EAPC − 5.2 for both), the economic burden of premature mortality due to childhood diarrhoeal diseases remained substantial. India accounted for the largest absolute loss in future labour income, estimated at USD 5,581 million PPP, followed by Pakistan (USD 1,571 million PPP) and Bangladesh (USD 360 million PPP). When expressed relative to national GDP using nominal estimates, Pakistan experienced the highest proportional economic loss, equivalent to approximately 0.055% of GDP in 2023, followed by India (0.020%), Bangladesh (0.012%), and Nepal (0.005%). Major etiological agents included rotavirus, Shigella, and Escherichia coli, while unsafe water, poor sanitation, and child undernutrition were the leading attributable risk factors.

**Conclusions and Policy Implications:**

Despite epidemiological gains, diarrhoeal diseases continue to impose substantial economic and developmental costs in South Asia. Strengthened investments in WASH infrastructure, nutrition, and vaccination programs can yield dual benefits by saving lives and reducing future productivity losses. Integrating child health strategies into national economic planning is imperative for sustainable growth.

**Supplementary Information:**

The online version contains supplementary material available at https://doi.org/10.1007/s44197-026-00598-9.

## Introduction

 Diarrhoeal diseases remain one of the leading causes of morbidity and mortality globally, accounting for nearly 1.5 million deaths each year, predominantly among children under five years of age [1]. Despite marked progress in sanitation, vaccination, and healthcare accessibility, diarrhoeal diseases continue to exert a heavy toll in low- and middle-income countries [2]. Their persistence significantly hampers progress toward achieving the Sustainable Development Goals (SDGs) related to health, clean water, and sanitation [2].

The South Asian region bears a disproportionate share of this global burden, contributing almost one-third of global childhood diarrhoeal deaths [3]. In India, diarrhoea affects approximately 8–12% of under-five children, while prevalence remains around 9% in Nepal and 11% in Bangladesh [4, 5]. Similar high rates have been reported in Pakistan and Bhutan, where diarrhoeal diseases continue to be major contributors to child mortality [6, 7]. The primary etiological agents include rotavirus, Shigella, enterotoxigenic Escherichia coli (ETEC), and Cryptosporidium, which together account for a large share of mortality and disability [1, 8].

Multiple risk factors contribute to the persistence of diarrhoeal diseases in South Asia. According to the Global Burden of Disease (GBD) 2021 estimates, nearly 60% of diarrhoeal deaths in children under five were attributable to unsafe water, sanitation, and hygiene (WASH) factors, and an additional 35–40% were linked to child undernutrition, including wasting and stunting [1]. Other significant determinants include low maternal education, inadequate breastfeeding practices, and unsafe food and water handling, which collectively amplify exposure to enteric pathogens [2, 3]. Climate-sensitive factors such as flooding and rising temperatures further elevate transmission risks, particularly in densely populated settlements [2].

Beyond its health burden, diarrhoea poses a substantial economic challenge. The annual societal cost of childhood diarrhoea exceeds US$80 million in Bangladesh [9], and rotavirus-related diarrhoea in India incurs losses of nearly US$200–250 million annually [8]. Pakistan and Nepal also face major household-level financial strain, with treatment costs consuming 2–3% of monthly income for low-income families [5, 6]. These studies mainly estimate direct treatment costs, household expenditures, and short-term financial hardship. Although useful for understanding the immediate burden on families and health systems, they do not capture the broader long-term economic consequences of premature child death, including lost future labour income, human capital, and national productivity. As a result, the full societal and developmental burden of under-five diarrhoeal mortality remains insufficiently understood in South Asia [10].

This study addresses this critical gap by quantifying lost future labour income resulting from premature mortality among children under five. Along with the annual percentage change in diarrhoeal disease burden in South Asia, analyzing age-specific mortality trends (2010–2023), identifying major aetiologies and risk factors.

## Methods

### Data

Data on deaths, probability of death, etiologies, and risk factors of diarrhoeal diseases were obtained from the datasets published by the Institute for Health Metrics and Evaluation (IHME) [11]. The IHME data were used instead of national datasets because they provide a standardized and internally consistent framework for estimating mortality, etiologies, risk factors, and uncertainty across countries and over time. This was particularly important for the present study, which aimed to undertake a comparable regional analysis across South Asian countries. The use of IHME estimates therefore allowed harmonized cross-country comparison using a common methodological approach.

Information on employment rates, GDP per capita in PPP-adjusted U.S. dollars (2023), and GDP per capita in nominal U.S. dollars (2023) was sourced from the World Bank’s World Development Indicators [12]. The annual productivity growth rate was assumed to be 1.5%, reflecting the long-term average growth in real GDP per worker across South Asian economies, consistent with regional economic performance trends reported by the World Bank [12, 13]. The average age at death due to diarrhoeal disease was not available; therefore, a mean age of 1 year was assumed for under-five mortality.

### Estimation of Loss of Future Labour Income

The economic loss associated with under-five diarrhoeal deaths was estimated using the human capital approach (HCA), which quantifies the present value of future labour income foregone due to premature mortality. This approach assumes that each under-five death results in the loss of all potential earnings the individual would have contributed to the economy from the expected age of entry into the workforce until the standard retirement age. This approach is appropriate for valuing long-term productivity losses from deaths occurring before labour-market entry. Alternative approaches, such as the friction cost approach and value of statistical life approach, were not applied because the former is more suited to short-term replacement-related productivity losses among current workers, while the latter requires context-specific willingness-to-pay estimates that were not consistently available across the included countries.

To provide a comprehensive assessment of economic loss, the analysis was performed using two complementary monetary approaches:

**Purchasing Power Parity (PPP)-adjusted U.S. dollars**, which capture the *real value* of lost productivity in terms of comparable purchasing power across countries, thus allowing for equitable cross-national comparison.

**Nominal U.S. dollars (market exchange rate)**, which reflect the *financial magnitude* of the loss in terms of the prevailing global currency value, thereby providing insight into the macroeconomic implications for each country’s economy and fiscal policy.

Using both PPP and nominal estimates allows the results to simultaneously represent the real productivity burden (PPP perspective) and the actual economic value of loss in financial terms (nominal perspective), ensuring both international comparability and national policy relevance.

### Assumptions Underlying the Estimation of Lost Future Labour Income

The average age at death due to diarrhoeal disease among children under five was not directly available in the dataset; therefore, a mean age at death of 1 year was assumed, reflecting the concentration of diarrhoeal mortality in infancy and early childhood. The age of entry into the workforce was set at 15 years to reflect the lower bound of working age commonly used in international labour statistics, while retirement age was assumed to be 65 years as a standard benchmark for estimating potential working life. Labour-force participation was incorporated using country-specific employment rates from the World Bank to avoid assuming that all individuals would be economically active.

### Formulas for Calculating the Total loss of Future Labour Income

Let.

- deathsU5 = number of under-five diarrhoeal deaths,
- W = gross domestic product (GDP) per worker (in PPP-USD or nominal USD).
- ER = employment (or labour-force participation) rate for the working-age population,
- E = age of entry into the workforce (assumed 15 years),
- A = retirement age (assumed 65 years),
- *r* = annual discount rate (base case 3%),
- *g* = annual productivity growth rate (assumed 1.5%), and.
- $\:{a}_{0}$= mean age at death among children under five (assumed 1 year).

The present value $\:{(PV}_{per\:death})\:$of lifetime earnings per death was computed as:

$$\:{PV}_{per\:death}=W\:\times\:ER\:\times\:\:\left(\frac{1-{q}^{N}}{1-q}\right)\:\times\:{(1+r)}^{-(E-\:{a}_{0}^{-})}\:$$

Where q = $$\:\frac{1+g}{1+r}$$ and *N = A – E + 1*.

The term $\:\frac{1-{q}^{N}}{1-q}$ represents the present value of a growing stream of future earnings *E* to *A*,

While $\:{(1+r)}^{-(E-\:{a}_{0})}$ discounts these earnings back to the age of death.

The total loss of future labour income due to under five diarrhoeal mortality then estimated as:

$$\:Loss=\:{deaths}_{U5}\:\times\:\:{PV}_{per\:death}$$

This approach implicitly assumes that, had the deceased children survived, they would have participated in the labour market at the average productivity level of current workers in their country. Estimates were expressed in both PPP-adjusted and nominal U.S. dollars. The 95% uncertainty intervals (UIs) for lost future labour income were derived by propagating the uncertainty bounds of diarrhoeal mortality estimates from IHME/GBD through the human capital model. Economic input parameters were treated as fixed in the base-case analysis; therefore, the reported UIs reflect uncertainty in mortality estimates only.

To assess robustness, we performed one-way deterministic sensitivity analyses by varying the discount rate (0%, 3%, and 5%), mean age at death (0.5, 1, and 2 years), annual productivity growth rate (1.0%, 1.5%, and 2.0%), and employment rate (base case ± 10%), while holding all other parameters constant as in base case model.

### Software

All graphical analyses including multiple bar charts with 95% UI, rankograms, heat maps, and radar chart along with the estimation of loss of future labour income due to under-five diarrhoeal mortality, were conducted using the R software version 4.5.1 [14].

## Results

In 2023, the epidemiological burden of diarrhoeal diseases in South Asia remained substantial, although it varied markedly across countries (Table 1). India accounted for the highest number of incident cases, DALYs, and deaths, reflecting both its large population and persistent disease burden. Pakistan ranked second for incidence and mortality, followed by Bangladesh, whereas Nepal and Bhutan contributed the smallest regional shares.

**Table 1:** Epidemiological burden of diarrhoeal diseases incidence, mortality and DALYs in South Asian countries in 2023

Geography	Incidence (95% UI) per 100,000	DALYs (95% UI) per 100,000	Mortality (95% UI) per 100,000	Incidence (95% UI) EAPC (1990–2023)	DALYs (95% UI) EAPC (1990–2023)	Mortality (95% UI) EAPC (1990–2023)
Bangladesh	41874.4 (34304.3 to 49770.1)	1314.8 (837.6 to 1908.8)	14 (8.7 to 20.7)	−3.3 (−3.5 to −3.1)	−8 (−8.7 to −7.3)	−6.5 (−7.2 to −5.9)
Bhutan	75,436 (62034.6 to 89868.2)	3803.3 (1860.9 to 7337.9)	41.3 (19.3 to 80.7)	−2.8 (−3.2 to −2.4)	−7.7 (−7.9 to −7.4)	−5.4 (−5.8 to −5)
India	88212.5 (71009.8 to 107487.8)	3019.3 (2272.4 to 3908.1)	32 (23.6 to 41.9)	−1.9 (−2.1 to −1.6)	−5.2 (−5.3 to −5.1)	−5.2 (−5.3 to −5)
Nepal	35485.5 (29440.5 to 41866.2)	1199.2 (605.7 to 2145.8)	12.8 (6.2 to 23.2)	−4.5 (−4.9 to −4.2)	−6.7 (−6.9 to −6.6)	−7.6 (−7.9 to −7.4)
Pakistan	113590.6 (89025.2 to 141428.9)	5316.3 (3072.9 to 8840.2)	57.5 (32.8 to 97.1)	−2.2 (−2.5 to −1.9)	−9.4 (−9.6 to −9.2)	−4.4 (−4.7 to −4.1)
South Asia	87478.7 (70687.4 to 106809.5)	3257.7 (2428.6 to 4282.1)	34.8 (25.7 to 46.2)	−5.2 (−5.3 to −5.1)	−4.8 (−5.2 to −4.4)	−5.2 (−5.3 to −5.1)
Global	69123.2 (56931.3 to 82041.3)	4501.2 (3076.2 to 6550.2)	49.2 (33.1 to 71.9)	−1.5 (−1.7 to −1.4)	−5.2 (−5.3 to −5.1)	−4.5 (−4.5 to −4.4)

Abbreviation: *DALY* Disability Adjusted Life Years, *UI* Uncertainty Intervals

Age-standardized estimates showed a sustained decline across all major indicators from 1990 to 2023, as reflected by negative estimated annual percentage changes (EAPCs). Bangladesh recorded the greatest annual reduction in DALYs (EAPC − 8.0) and a marked decline in mortality (EAPC − 6.5). Nepal also showed rapid improvement, particularly in mortality (EAPC − 7.6) and DALYs (EAPC − 6.7). Pakistan experienced the steepest decline in DALYs (EAPC − 9.4), although its reduction in mortality was more moderate (EAPC − 4.4). In contrast, India showed smaller but consistent declines in incidence (EAPC − 1.9), DALYs (EAPC − 5.2), and mortality (EAPC − 5.2). Bhutan maintained the lowest absolute burden throughout the region, with steady reductions across all parameters.

At the regional level, South Asia showed faster declines in incidence and mortality than the global average, with EAPCs of − 5.2 for both indicators. Overall, these findings indicate substantial progress in reducing the burden of diarrhoeal diseases over the past three decades, despite persistent cross-country differences.

**Fig. 1:**
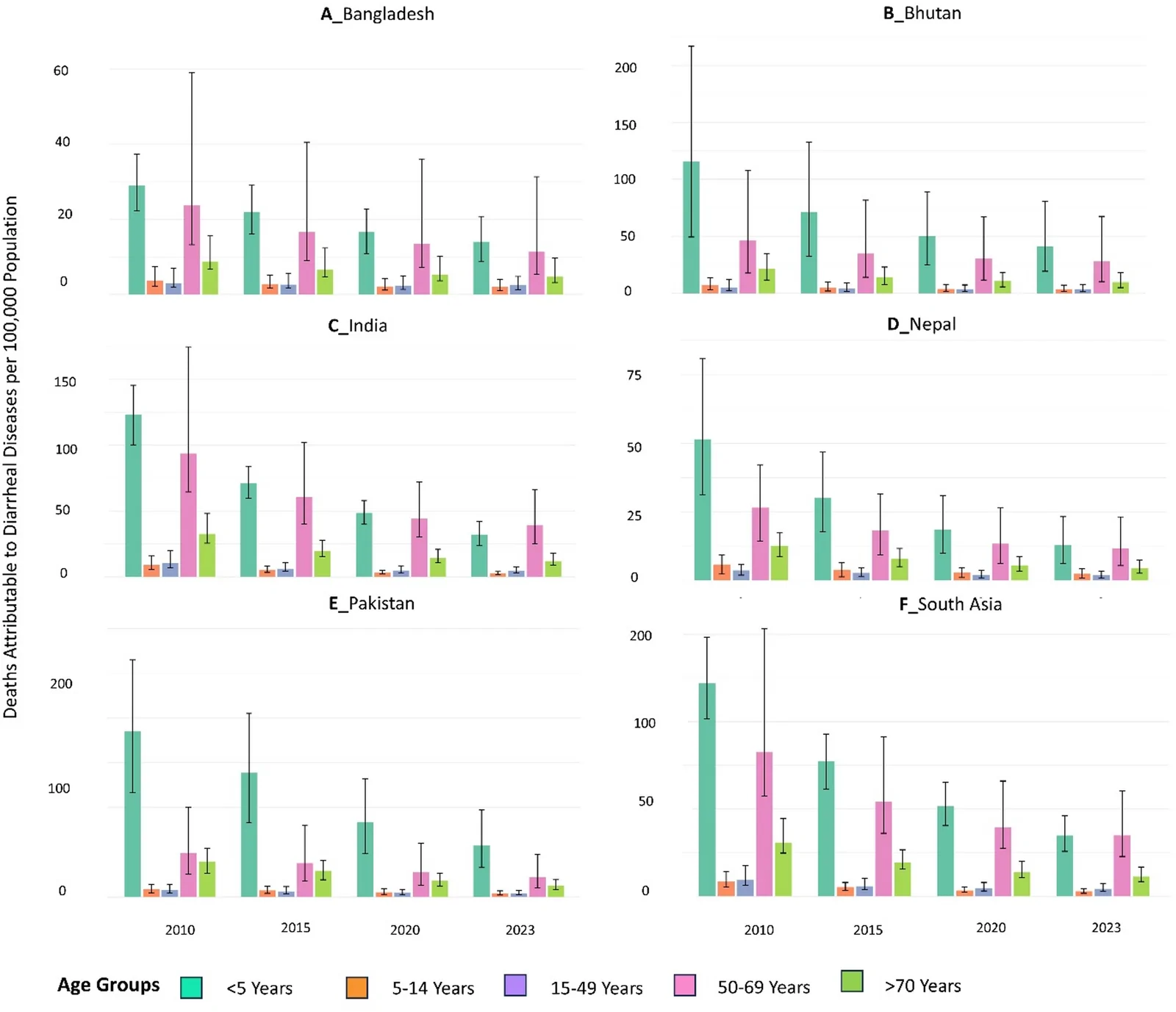
Age-Standardized Death Rate (per 100,000 Population, 95% UI) Attributable to Diarrhoeal Diseases in South Asia Among Individuals Aged 0–70 Years, 2010–2023

Age-standardized death rates attributable to diarrhoeal diseases declined consistently across South Asia between 2010 and 2023, although the magnitude of reduction varied by country (Fig. 1). Children under five years continued to bear the highest mortality burden. Adults aged 50–69 years represented the second most affected age group, indicating persistent vulnerability at both extremes of the age spectrum.

India and Pakistan showed the largest absolute declines over the study period, with under-five mortality falling by more than half since 2010. Bangladesh and Nepal also demonstrated steady improvement across all age groups. In contrast, Bhutan maintained relatively low mortality rates, although year-to-year variation was more apparent.

Despite these encouraging declines, mortality remained measurable among both young children and older adults. This pattern suggests that diarrhoeal diseases continue to represent an important public health concern in South Asia.

**Fig. 2:**
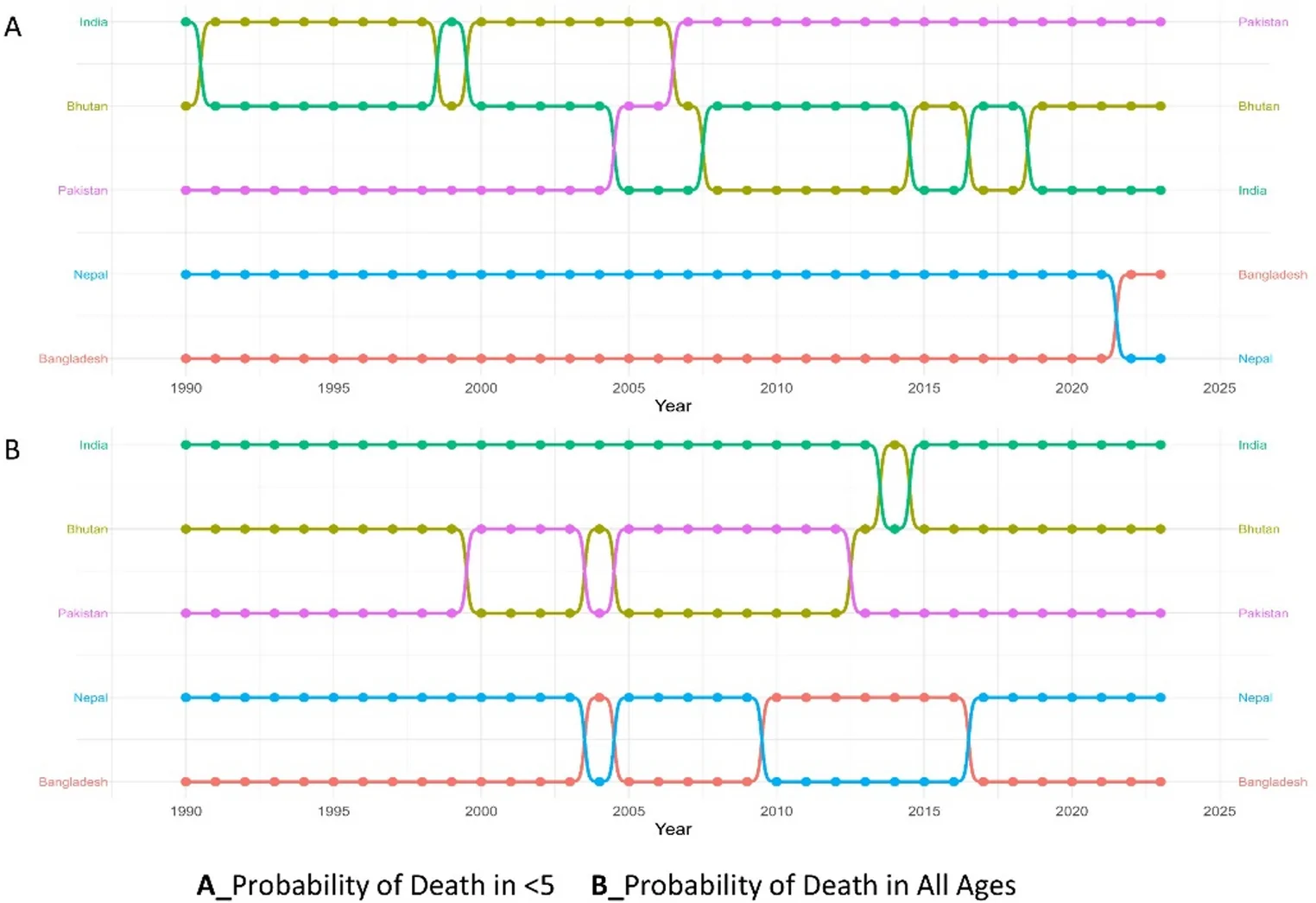
Relative Country Rankings for Probability of Death Due to Diarrhoeal Diseases in South Asia, 1990–2023: (**A**) Children Under 5 Years of Age; (**B**) All Age Groups

Over the period from 1990 to 2023, the relative country ranking of probability of death due to diarrhoeal diseases showed distinct patterns across South Asia Fig. 2. Among children under five years, Bhutan and India occupied higher relative positions during the early years, while Pakistan moved to the highest relative position from the mid-2000s onward and remained there through 2023. India shifted from a higher relative position in the early 1990 s to an intermediate position in the later years. Bangladesh and Nepal generally remained in the lower relative positions, although Bangladesh moved above Nepal toward the end of the study period.

 For all age groups combined, India remained in the highest relative position for most of the study period. Bhutan and Pakistan largely occupied intermediate positions, with some rank changes over time. Bangladesh and Nepal remained in the lower relative positions, with occasional exchanges in rank during the study period.

 Overall, Fig. 2 indicates that the comparative burden of diarrhoeal mortality did not shift uniformly across South Asia. Pakistan remained relatively more affected among children under five in the later years, while India continued to occupy a higher relative position when all age groups were considered. These ranking patterns suggest persistent country-level heterogeneity in diarrhoeal mortality risk, even as absolute mortality rates declined across the region as shown in the rate-based estimates.

**Fig. 3:**
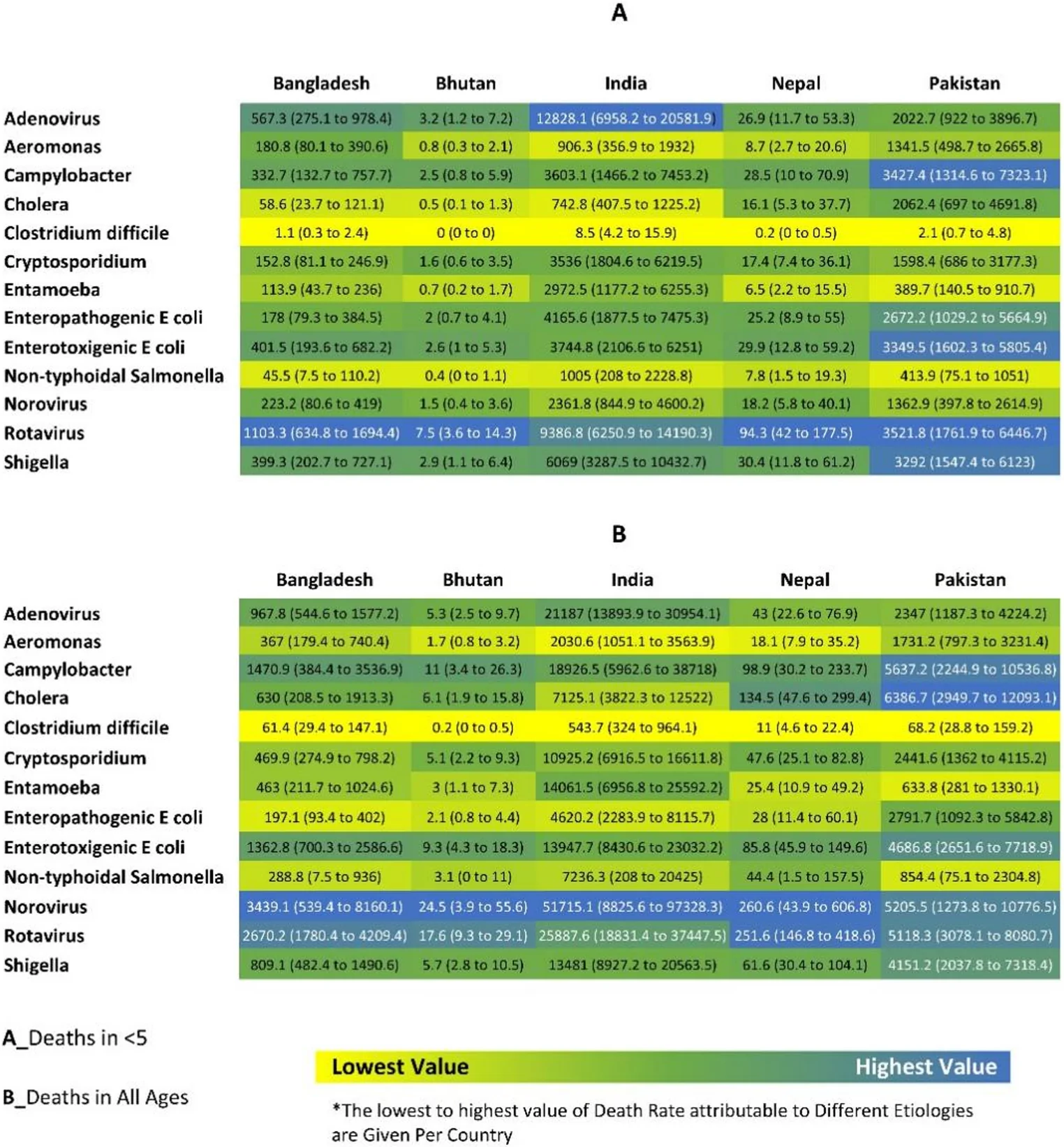
Death Rate (per 100,000 Population, 95% UI) Attributable to Different Aetiologies of Diarrhoeal Diseases in South Asia, 2023: (**A**) Children Under 5 Years of Age; (**B**) All Age Groups

Mortality patterns by aetiology further highlighted important differences across South Asian countries (Fig. 3). In 2023, the leading causes of under-five diarrhoeal mortality varied by country. In India, Rotavirus, *Shigella*, and enterotoxigenic and enteropathogenic *E. coli* were the most prominent pathogens. In Pakistan, Rotavirus and Cholera were the dominant causes. In Bangladesh, Rotavirus remained the leading cause, followed by Norovirus and *Shigella*. Nepal and Bhutan had lower overall mortality levels, although *Cryptosporidium* and *Campylobacter* accounted for a relatively greater share of deaths in these countries.

 A similar but not identical pattern was observed when all age groups were considered. India continued to show the highest pathogen-specific mortality, particularly from *Campylobacter*, Rotavirus, enterotoxigenic *E. coli*, and *Shigella*. Pakistan showed a broadly comparable profile, whereas Bangladesh remained characterized by the predominance of Rotavirus and Norovirus. Nepal and Bhutan again recorded the lowest overall pathogen-specific mortality.

 Overall, Rotavirus remained the leading cause of under-five diarrhoeal mortality across the region. In contrast, among all age groups, *Campylobacter* and *E. coli* species contributed a relatively larger share of mortality.

**Fig. 4:**
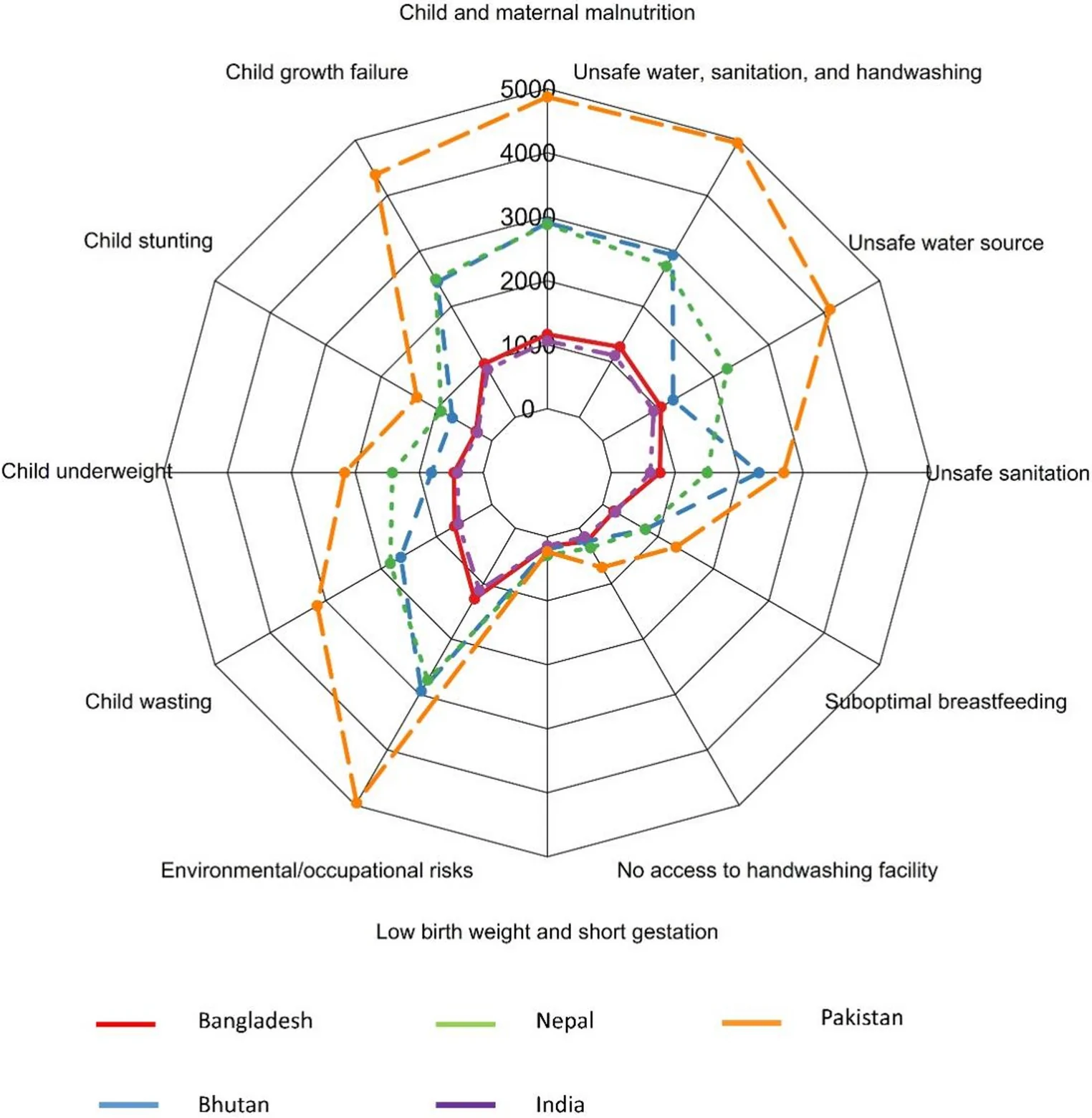
Disability-adjusted life years (DALYs) per 100,000 population attributable to different risk factors for diarrhoeal diseases in South Asia, 2023

Assessment of disability-adjusted life years (DALYs) attributable to major risk factors revealed clear cross-country differences in South Asia (Fig. 4). Risk-factor contributions were presented using the GBD-defined categories as reported, with each estimate reflecting the attributable burden for that specific risk domain in the GBD framework. Pakistan had the highest DALY burden overall, with the largest contributions from unsafe water, sanitation, and handwashing, as well as child and maternal malnutrition, followed by child growth failure. India also showed a substantial burden, although it was more evenly distributed across multiple risks. The leading contributors in India were unsafe water source, unsafe sanitation, and child growth failure.

 Bangladesh occupied an intermediate position, with suboptimal breastfeeding and child growth failure emerging as the main contributors. Nepal showed comparatively lower DALY levels, but unsafe water source and child growth failure remained prominent. Bhutan had the lowest overall burden, with only modest elevations related to unsafe water, sanitation, and handwashing.

 Overall, unsafe water and sanitation and child undernutrition-related factors were the leading contributors to diarrhoeal disease burden across South Asia. However, their relative importance varied across countries.

The estimated loss of future labour income due to under-five diarrhoeal mortality in South Asia in 2023 varied substantially across countries. Using GDP per capita expressed in PPP adjusted international dollars, the analysis captured the real productivity loss after accounting for differences in purchasing power and cost of living. This approach allowed more comparable cross-country assessment of the economic burden.

At a 3% discount rate, India experienced the highest estimated loss of future labour income attributable to premature mortality from childhood diarrhoeal diseases, amounting to USD 5,581 million PPP (95% UI: 4,107–7,306 million). Pakistan ranked second, with estimated losses of USD 1,571 million PPP (95% UI: 896–2,653 million), followed by Bangladesh at USD 360 million PPP (95% UI: 223–534 million). Nepal and Bhutan incurred comparatively smaller losses, estimated at USD 20 million PPP (95% UI: 10–37 million) and USD 6 million PPP (95% UI: 2–12 million), respectively (Table 2).

**Table 2:** Estimated loss of future labour income attributable to premature mortality among children due to diarrhoeal diseases in South Asia

Country	Lost future labour income due to premature mortality in the year 2023		
	At Discount rate 3%	At Discount rate 0%	At Discount rate 5%
In Million PPP-USD (95% UI)			
Bangladesh	360 (223 to 534)	1142 (709 to 1692)	187 (116 to 278)
Bhutan	6 (2 to 12)	20 (9 to 39)	3 (1 to 6)
India	5581 (4107 to 7306)	17,689 (13017 to 23156)	2908 (2140 to 3808)
Nepal	20 (10 to 37)	65 (31 to 119)	10 (5 to 19)
Pakistan	1571 (896 to 2653)	4979 (2841 to 8410)	818 (467 to 1383)
	In Million Nominal USD (95% UI)		
Bangladesh	52 (32 to 77)	318 (197 to 472)	100 (62 to 148)
Bhutan	0 (0 to 1)	4 (2 to 9)	1 (0 to 2)
India	712 (524 to 933)	4335 (3190 to 5675)	1367 (1006 to 1790)
Nepal	2 (1 to 5)	16 (8 to 30)	5 (2 to 9)
Pakistan	185 (105 to 312)	1126 (642 to 1902)	355 (202 to 600)

Abbreviation: *GDP* Gross domestic product, *UI* Uncertainty intervals, *USD* US Dollar, *PPP* Purchasing power parity

 When expressed in nominal U.S. dollars, India again accounted for the largest economic burden, with estimated losses of USD 712 million (95% UI: 524–933 million), equivalent to 0.020% of national GDP in 2023. Pakistan followed with losses of USD 185 million (95% UI: 105–312 million), representing 0.055% of GDP, while Bangladesh recorded losses of USD 52 million (95% UI: 32–77 million), equivalent to 0.012% of GDP. In Nepal, estimated losses amounted to USD 2 million (95% UI: 1–5 million), corresponding to approximately 0.005% of GDP. Bhutan experienced the lowest nominal losses, estimated at less than USD 1 million.

Expressing these losses as a proportion of GDP highlights the macroeconomic importance of preventable under-five mortality, especially in low- and middle-income settings where even modest absolute losses can reduce future productive capacity. Taken together, the PPP-adjusted and nominal estimates provide complementary perspectives on the economic burden. PPP-adjusted values reflect real productivity losses in internationally comparable terms, whereas nominal estimates capture the fiscal significance of those losses within each country’s economy. Overall, the findings reinforce the economic case for investing in child survival, sanitation, and preventive health systems. Reducing under-five diarrhoeal mortality would not only save lives but also help avert substantial future productivity losses and support long-term economic stability in the region.

One-way sensitivity analyses (Table S1) demonstrated that the absolute estimates of lost future labour income varied under alternative assumptions, as expected. However, the overall pattern of country-level findings remained unchanged, with India consistently showing the largest economic loss, followed by Pakistan and Bangladesh. These results support the robustness of the main conclusions.

## Discussion

The primary objective of this study was to quantify the economic losses associated with premature under-five mortality due to diarrhoeal diseases in South Asia, applying the human capital approach to estimate the value of lost future labour income. The findings indicate a marked decline in diarrhoeal mortality and disability over the past decade, yet the persistence of substantial productivity losses highlights the ongoing developmental impact of child mortality in the region. Despite epidemiological progress, the economic burden remains disproportionately high, particularly in countries with large populations and variable access to clean water and sanitation.

Recent analyses indicate that, although the burden of diarrhoeal diseases has declined substantially since 1990, children under five continue to account for a major share of mortality and disability, particularly in low- and middle-income settings [1]. A recent South Asia-specific population-based analysis also reported persistent childhood diarrhoeal burden with marked heterogeneity across countries, which is consistent with the cross-country variation observed in our study [3]. The predominance of Rotavirus, *Shigella*, and *Escherichia coli* in our results is broadly consistent with earlier multicountry aetiological evidence from the Global Enteric Multicenter Study (GEMS), which identified rotavirus, *Shigella*, enterotoxigenic *E. coli*, and Cryptosporidium among the leading causes of moderate-to-severe diarrhoeal disease in children under five in Africa and South Asia [15]. Likewise, the prominent contribution of unsafe water, sanitation, and child undernutrition in our analysis aligns with GBD-based risk-factor estimates and with meta-analytic evidence showing that WASH interventions reduce childhood diarrhoeal risk [1, 16]. Emerging evidence further suggests that increases in temperature and rainfall can elevate childhood diarrhoeal risk, particularly in settings with unimproved water and sanitation, which may help explain the persistence of disease burden in vulnerable populations [17].

The persistence of WASH-related and nutritional determinants underscores the need for interventions that directly target the leading risks identified in our study. A recent systematic review and meta-analysis confirmed that improvements in drinking water, sanitation, and handwashing reduce childhood diarrhoeal risk in low- and middle-income settings, supporting continued investment in high-quality WASH infrastructure and hygiene promotion [16]. This is particularly relevant in climate-sensitive settings, as recent evidence shows that temperature and rainfall variability can increase childhood diarrhoeal risk [17]. In rural Bangladesh, a recent analysis of the WASH Benefits trial further showed that improved household WASH had the largest benefits among lower socioeconomic groups and during the monsoon season; scaling up a similar intervention was estimated to prevent 734 (95% CI 385–1085) diarrhoeal cases per 1000 children per month during the seasonal monsoon [18]. Because child undernutrition was also a major contributor in our analysis, nutrition-sensitive interventions remain highly relevant. The World Bank’s *Investment Framework for Nutrition 2024* estimated that scaling up high-impact nutrition interventions could avert 6.2 million under-five deaths over the next decade and generate US$2.4 trillion in economic benefits globally, equivalent to an estimated return of US$23 for every US$1 invested [19]. Likewise, given the prominent contribution of rotavirus in our etiological analysis, strengthening rotavirus vaccination is likely to be economically justified. In Bangladesh, a recent economic evaluation projected that universal childhood rotavirus vaccination could prevent about 1.54 million cases within the first 2 years and generate a benefit-cost ratio of about 2.03–2.20 depending on delivery strategy [20]. Taken together, these findings suggest that integrating WASH, nutrition, vaccination, and climate-resilient planning into child health policy could reduce preventable mortality while also yielding substantial long-term economic gains through avoided productivity losses.

From an economic perspective, this study extends the discourse by framing diarrhoeal mortality as a loss of national productivity rather than merely a healthcare cost. Recent evaluations using the human capital and value-of-statistical-life (VSL) frameworks emphasize that preventable mortality imposes long-term economic constraints through diminished labour participation and productivity [21, 22]. For example, cross-country analyses of child mortality–linked productivity losses estimate annual global losses exceeding US$100 billion in Africa [23]. While previous household cost-of-illness studies capture immediate financial strain, macroeconomic assessments such as the present one reveal the intergenerational implications of lost human capital.

This study provides important evidence on the magnitude of economic losses associated with premature under-five mortality due to diarrhoeal diseases in South Asia by integrating epidemiological and economic data to show how preventable child deaths translate into future productivity deficits. Nevertheless, several limitations should be acknowledged. First, the analysis relies primarily on IHME estimates, which are modelled from multiple data sources of varying quality and completeness. These modelled estimates may not fully capture recent local variation in mortality, aetiology, or risk-factor patterns, particularly in settings with limited primary data [24]. Second, the human capital approach is based on simplifying assumptions and may overestimate productivity losses because it assumes that future labour income lost due to premature death is not replaced in the labour market. Although country-specific employment rates were incorporated, the model applies average labour-force participation, GDP per worker, and productivity growth, and therefore may not reflect informal work, unemployment, changing labour-market conditions, or future economic dynamics [22, 25]. Third, the uncertainty intervals reflect uncertainty in mortality estimates only, while economic parameters were treated as fixed; therefore, the intervals may not capture the full uncertainty around the economic estimates. Fourth, the analysis is restricted to mortality-related productivity losses and does not include non-fatal morbidity, long-term developmental consequences, caregiver time losses, or unpaid household labour [22, 26]. As a result, the total societal burden of diarrhoeal diseases in young children is likely to be underestimated. Finally, the estimates are presented at the national level and do not capture within-country heterogeneity, such as rural–urban, regional, or socioeconomic disparities, which may limit their direct use for highly targeted subnational policy planning. Despite these limitations, the estimates provide a useful regional perspective on the macroeconomic consequences of preventable under-five diarrhoeal mortality in South Asia.

## Conclusion

Despite substantial declines in mortality, diarrhoeal diseases continue to impose an important economic burden on South Asian countries through preventable under-five deaths. The productivity losses estimated in this study suggest that diarrhoeal mortality remains relevant not only as a public health concern but also as a developmental challenge. These findings support the need for continued efforts to improve WASH infrastructure, child nutrition, vaccination coverage, and climate-resilient public health planning. Although the present analysis did not directly evaluate specific interventions, reducing under-five diarrhoeal mortality would likely contribute to both improved child survival and lower long-term productivity losses. Integrating child health priorities into broader development planning may therefore support progress toward both health and economic goals in the region.

## Supplementary Information

Below is the link to the electronic supplementary material.

Supplementary Material 1 (DOCX 18.4 KB)

## Data Availability

All the data used in this study are available online at the Institute of health metrics and evaluation’s website and World Bank’s data bank.

## References

[1] Kyu HH, Vongpradith A, Dominguez R-M, Ma J, Albertson SB, Novotney A, et al. Global, regional, and national age-sex-specific burden of diarrhoeal diseases, their risk factors, and aetiologies, 1990–2021, for 204 countries and territories: a systematic analysis for the Global Burden of Disease Study 2021. Lancet Infect Dis. 1990;25(5):519–36. 10.1016/s1473-3099(24)00691-1. PMC12018300 39708822

[2] Alum EU, Obeagu EI, Ugwu O-C. Enhancing quality water, good sanitation, and proper hygiene is the panacea to diarrhea control and the attainment of some related sustainable development goals: a review. Medicine (Baltimore). 2024. 10.1097/md.0000000000039578. 39312342 PMC11419503

[3] Sarker AR, Hasan A, Nawar N, Islam R. Childhood diarrhoeal disease in South Asian countries: a population-based analysis. BMJ Public Health. 2025. 10.1136/bmjph-2024-001909. 40791273 PMC12336502

[4] Ghosh K, Chakraborty AS, Mog M. Prevalence of diarrhoea among under five children in India and its contextual determinants: a geo-spatial analysis. Clin Epidemiol Glob Health. 2021. 10.1016/j.cegh.2021.100813.

[5] Li R, Lai Y, Feng C, Dev R, Wang Y, Hao Y. Diarrhea in under five year-old children in Nepal: a spatiotemporal analysis based on demographic and health survey data. Int J Environ Res Public Health. 2020. 10.3390/ijerph17062140. 32210171 PMC7142451

[6] Rahmat ZS, Zubair A, Abdi I, Humayun N, Arshad F, Essar MY. The rise of diarrheal illnesses in the children of Pakistan amidst COVID-19: a narrative review. Health Sci Rep. 2023. 10.1002/hsr2.1043. 36620511 PMC9811062

[7] Wangdi K, Clements ACA. Spatial and temporal patterns of diarrhoea in Bhutan 2003–2013. BMC Infect Dis. 2017. 10.1186/s12879-017-2611-6. 28732533 PMC5521140

[8] Tate JE, Chitambar S, Esposito DH, Sarkar R, Gladstone B, Ramani S, et al. Disease and economic burden of rotavirus diarrhoea in India. Vaccine. 2009;27:F18–24. 10.1016/j.vaccine.2009.08.098. 19931713

[9] Hasan MZ, Mehdi GG, De Broucker G, Ahmed S, Ali MW, Martin Del Campo J, et al. The economic burden of diarrhea in children under 5 years in Bangladesh. Int J Infect Dis. 2021;107:37–46. 10.1016/j.ijid.2021.04.038. 33864914 PMC8208894

[10] Al Fidah MF, Islam MR, Amin R, Nuzhat S, Ahmed T, Faruque ASG. The cost of diarrhoea: a household perspective from seven countries in the Global Enteric Multicenter Study (GEMS). BMJ Paediatr Open. 2025. 10.1136/bmjpo-2025-003622. 40670046 PMC12273133

[11] Results IHMEGBD. 2025 [Available from: https://vizhub.healthdata.org/gbd-results/

[12] Bank W. World Development Indicators 2025 [Available from: https://databank.worldbank.org/reports.aspx?source=2&series=NY.GDP.PCAP.CD&country=#.

[13] Young Eun Kim NVL. Productivity Growth Patterns and Determinants across the World. 2019.

[14] The R Project for Statistical Computing. 2025 [Available from: https://www.r-project.org/

[15] Kotloff KL, Nataro JP, Blackwelder WC, Nasrin D, Farag TH, Panchalingam S, et al. Burden and aetiology of diarrhoeal disease in infants and young children in developing countries (the Global Enteric Multicenter Study, GEMS): a prospective, case-control study. Lancet. 2013;382(9888):209–22. 10.1016/s0140-6736(13)60844-2. 23680352

[16] Wolf J, Hubbard S, Brauer M, Ambelu A, Arnold BF, Bain R, et al. Effectiveness of interventions to improve drinking water, sanitation, and handwashing with soap on risk of diarrhoeal disease in children in low-income and middle-income settings: a systematic review and meta-analysis. Lancet. 2022;400(10345):48–59. 10.1016/s0140-6736(22)00937-0. 35780792 PMC9251635

[17] Geremew G, Cumming O, Haddis A, Freeman MC, Ambelu A. Rainfall and temperature influences on childhood diarrhea and the effect modification role of water and sanitation conditions: a systematic review and meta-analysis. Int J Environ Res Public Health. 2024. 10.3390/ijerph21070823. 39063400 PMC11276699

[18] Ante-Testard PA, Rerolle F, Nguyen AT, Ashraf S, Parvez SM, Naser AM, et al. WASH interventions and child diarrhea at the interface of climate and socioeconomic position in Bangladesh. Nat Commun. 2024;15(1):1556. 10.1038/s41467-024-45624-1. 38378704 PMC10879131

[19] Bank W. Investment Framework for Nutrition 2024. Washington, DC: World Bank; 2024.

[20] Sarker AR. Economic assessment of childhood rotavirus vaccination in Bangladesh. J Infect Public Health. 2023;16(5):816–22. 10.1016/j.jiph.2023.03.021. 37003027

[21] Scott A, Ashwin J, Ellison M, Sinclair D. International gains to achieving healthy longevity. Cold Spring Harb Perspect Med. 2023. 10.1101/cshperspect.a041202. 36096548 PMC9899644

[22] Krol M, Brouwer W, Rutten F. Productivity costs in economic evaluations: past, present, future. Pharmacoeconomics. 2013;31(7):537–49. 10.1007/s40273-013-0056-3. 23620213

[23] Kirigia JM, Muthuri RDK, Nabyonga-Orem J, Kirigia DG. Counting the cost of child mortality in the World Health Organization African region. BMC Public Health. 2015. 10.1186/s12889-015-2465-z. 26545350 PMC4636778

[24] Jewell NP, Lewnard JA, Jewell BL. Caution warranted: using the Institute for Health Metrics and Evaluation model for predicting the course of the COVID-19 pandemic. Ann Intern Med. 2020;173(3):226–7. 10.7326/m20-1565. 32289150 PMC7197035

[25] Pike J, Grosse SD. Friction cost estimates of productivity costs in cost-of-illness studies in comparison with human capital estimates: a review. Appl Health Econ Health Policy. 2018;16(6):765–78. 10.1007/s40258-018-0416-4. 30094591 PMC6467569

[26] Donowitz JR, Drew J, Taniuchi M, Platts-Mills JA, Alam M, Ferdous T, et al. Diarrheal pathogens associated with growth and neurodevelopment. Clin Infect Dis. 2021;73(3):e683–91. 10.1093/cid/ciaa1938. 33399861 PMC8326554

