# Supplementary material for: Economic Losses from Premature Under-Five Mortality due to Diarrhoeal Diseases in South Asia: Trends, Aetiologies, and Key Risk Factors
Source: J Epidemiol Glob Health. 2026 Jul 3;16(1):109. doi: 10.1007/s44197-026-00598-9 (PMC13558523; doi:10.1007/s44197-026-00598-9)
Supplement: Supplementary file 1 [file 44197_2026_598_MOESM1_ESM.docx]

**Supplementary Table S1:** One-way sensitivity analysis of estimated loss of future labour income due to under-five diarrhoeal mortality in South Asia, 2023

| **Country** | **Base case point estimate** | **Age 0.5 y** | **Age 2 y** | **g=1.0%** | **g=2.0%** | **ER -10%** | **ER +10%** | **Change in conclusion** |
| --- | --- | --- | --- | --- | --- | --- | --- | --- |
| **In Million PPP-USD** | | | | | | | | |
| Bangladesh |  | 357.9346976 | 365.8984535 | 302.8377174 | 431.1324985 | 299.4563038 | 421.6833666 | No |
| Bhutan |  | 6.283228269 | 6.423024987 | 5.316049322 | 7.568151175 | 5.324805504 | 7.334166072 | No |
| India |  | 5540.706411 | 5663.982622 | 4687.824045 | 6673.783275 | 4508.132547 | 6654.862331 | No |
| Nepal |  | 20.63773735 | 21.09691022 | 17.46096512 | 24.85816359 | 15.01476432 | 26.56458302 | No |
| Pakistan |  | 1559.641952 | 1594.342717 | 1319.565864 | 1878.589408 | 1256.899307 | 1885.348961 | No |
| **In Million Nominal USD** | | | | | | | | |
| Bangladesh |  | 99.81635015 | 102.0371828 | 100.5512044 | 120.228837 | 83.50862736 | 117.5937814 | No |
| Bhutan |  | 1.484604755 | 1.517636003 | 1.495534508 | 1.788207072 | 1.258148078 | 1.732920938 | No |
| India |  | 1357.936166 | 1388.149141 | 1367.933377 | 1635.634701 | 1104.869266 | 1630.997488 | No |
| Nepal |  | 5.287687504 | 5.405334251 | 5.326615791 | 6.369021892 | 3.847000293 | 6.806231288 | No |
| Pakistan |  | 352.780564 | 360.6296446 | 355.3777566 | 424.9243423 | 284.3022053 | 426.4533079 | No |

**y:**years, **g:**annual productivity growth rate, **ER:** employment rate for the working-age population
